# Diagnosis and Management of Chronic Nonbacterial Osteomyelitis in a Dog

**DOI:** 10.3390/ani15111593

**Published:** 2025-05-29

**Authors:** Young-Sun Jeong, Yun-Joo Geum, Hyun-Jung Han

**Affiliations:** 1 Department of Veterinary Emergency and Critical Care Medicine, College of Veterinary Medicine, Konkuk University, Seoul 05029, Republic of Korea; tjsdl96@konkuk.ac.kr (Y.-S.J.);; 2 KU Center for Animal Blood Medical Science, Konkuk University, Seoul 05029, Republic of Korea

**Keywords:** bisphosphonates, canine, disease-modifying anti-rheumatic drugs (DMARDs), glucocorticoids, non-infectious osteomyelitis

## Abstract

This case presents the first documented successful medical management of chronic nonbacterial osteomyelitis (CNO) in a small dog—a condition rarely described in veterinary medicine. A 4-year-old castrated male Pomeranian dog weighing 4.6 kg was presented with a 3-week history of right hindlimb lameness. Radiographic examination revealed osteolytic lesions, periosteal reactions, and decreased muscle mass in the affected limb. Microbial and blood culture tests showed negative results, whereas cytological and histopathological analyses of the right distal femur confirmed neutrophilic inflammation and osteomyelitis, leading to a diagnosis of CNO. Clinical and radiographic improvements were observed following administration of bisphosphonates, disease-modifying anti-rheumatic drugs, and glucocorticoids. This case underscores the possibility of non-infectious osteomyelitis in dogs and suggests that adapting human treatment strategies may be beneficial. Further research is needed to clarify the diagnostic criteria and pathophysiology of CNO in veterinary medicine.

## Simple Summary

Chronic nonbacterial osteomyelitis (CNO) is a rare inflammatory bone condition in animals. This report presents the first veterinary case of CNO successfully managed with medical therapy alone, using anti-inflammatory and immunomodulatory drugs adapted from human protocols. The dog showed clinical and radiographic improvement after treatment. These findings suggest that non-infectious osteomyelitis can occur in dogs and highlight the potential applicability of human treatment strategies in veterinary medicine.

## 1. Introduction

Osteomyelitis is an inflammatory bone disease commonly caused by microbial infection [1], and in rare cases, osteomyelitis can occur without infection—known as chronic nonbacterial osteomyelitis (CNO) [2]. Chronic recurrent multifocal osteomyelitis (CRMO) is considered a severe form of CNO, characterized by chronically active or recurrent inflammation of multiple bones [3]. Although the exact pathophysiology of CNO/CRMO in humans has not been fully determined, it is believed to be an autoimmune disease, stemming from cytokine dysregulation and osteoclast activation, which accelerate bone remodeling and inflammatory bone damage [3,4].

In humans, a general treatment guideline for CNO affecting the spine has been established and applied to other CNO patients, with slight variations focusing on bone-related pain relief and immunomodulation [5]. Nonsteroidal anti-inflammatory drugs (NSAIDs) are commonly chosen as first-line agents, providing quick symptomatic relief [5]. Other agents such as bisphosphonates, disease-modifying anti-rheumatic drugs (DMARDs), and glucocorticoids have been reported to be effective in small cohorts of CNO patients [5].

In humans, CNO is rare and accounts for approximately 2–5% of all osteomyelitis cases [2]. Similarly, CNO is very uncommon in veterinary medicine, with only three reported cases [6,7,8]. Notably, unlike the treatment methods commonly used in humans, those veterinary cases were managed with reconstructive surgery and administering antibiotics alongside NSAIDs [6,7,8]. To the authors’ knowledge, this is the first documented case in which a dog with CNO was successfully treated using bisphosphonates, DMARDs, and glucocorticoids.

## 2. Case Description

A 4-year-old castrated male Pomeranian dog weighing 4.6 kg with a 3-week history of right hindlimb lameness was referred to the Department of Emergency and Critical Care at Konkuk University Veterinary Medical Teaching Hospital. The referring veterinarian had prescribed an NSAID, specifically meloxicam (0.2 mg/kg PO q24h), for 2 weeks; however, the lameness did not improve. Anorexia and lethargy had developed 2 days prior to presentation. Bilateral patellar surgery was performed two years ago without any complications or clinical signs and is therefore considered unlikely to be associated with the current lameness.

Upon physical examination, the rectal temperature was 40.3 °C, and the respiratory rate was 60 breaths/min; otherwise, all findings were within normal limits. Gait assessment revealed non-weight-bearing lameness of the right hindlimb (Supplementary Video S1), and pain was observed upon palpation of the right stifle joint region. The dog was bright, alert, and responsive, with no other abnormal clinical findings.

**Figure 1:**
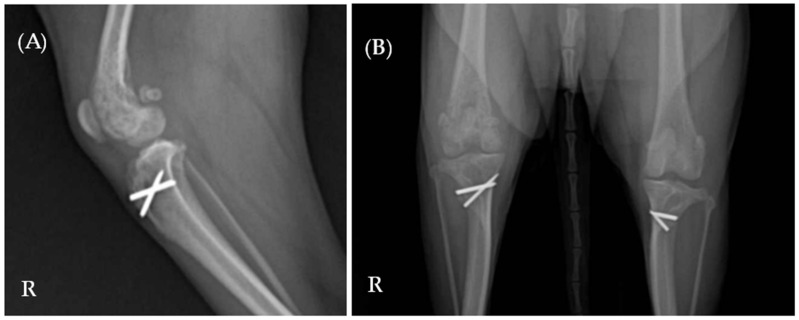
(**A**) Mediolateral and (**B**) ventrodorsal radiographic views of the right hindlimb. Multifocal lesions are evident in both the right proximal tibia and distal femur. The letter ‘R’ indicates the right side of the patient.

Laboratory results indicated elevated levels of C-reactive protein (CRP) (9.5 mg/dL; reference range 0.1–1.0 mg/dL) and alkaline phosphatase (ALP) (319 U/L; reference range 23–212 U/L). Complete blood count and serum biochemistry, including Albumin (2.4 g/dL [2.3–4.0 g/dL]), Alanine Aminotransferase (ALT) (28 U/L [10–125 U/L]), Blood Urea Nitrogen (BUN) (12 mg/dL [7–27 mg/dL]), Creatinine (1.2 mg/dL [0.5–1.8 mg/dL]), Glucose (94 mg/dL [74–143 mg/dL]), and Total Protein (7.6 g/dL [5.2–8.2 g/dL]) were all confirmed to be within the normal range. Radiographic imaging identified multifocal lesions in the right hindlimb, including osteolysis of the right proximal tibia, a periosteal reaction in the right distal femur, and increased opacity within the right knee joint space (Figure 1). Additionally, reduced muscle thickness was noted at the mid-femur region on the right side compared to the left.

For histopathologic examination, a bone biopsy was performed using a 16-G Jamshidi needle (Argon Medical Devices, Plano, TX, USA) under general anesthesia, and synovial fluid was collected via arthrocentesis. All samples were submitted for cytological, histopathological, aerobic, anaerobic, and fungal culture tests. Post-biopsy radiographs of the right hindlimb confirmed no iatrogenic fractures or complications, and the dog was discharged after 1 day of sample collection.

**Figure 2:**
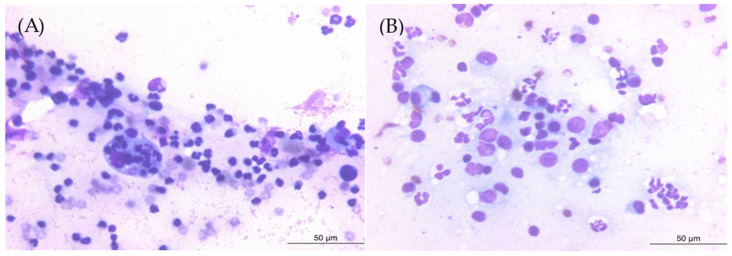
Cytology of the right hindlimb lesion. (**A**) Hypersegmented neutrophils with no evidence of pathogens or phagocytosis in the synovial fluid. (**B**) Infiltration of neutrophils and plasma cells in the distal femur bone tissue.

**Figure 3:**
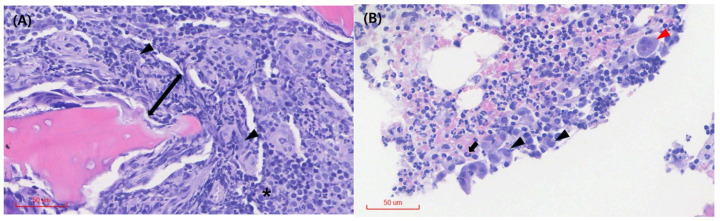
Histopathology of the right distal femur bone tissue. (**A**) Tissue necrosis (arrow) is observed around the osseous (or cartilaginous) tissue, and the medullary spaces are primarily infiltrated by moderate numbers of histiocytes (arrowhead). Plasma cells (*) are visible. (**B**) Multinucleated giant cells (red arrowhead), degenerated neutrophils (black arrow), and histiocytes (black arrowhead) are observed among the inflammatory cell infiltrates. No obvious evidence of neoplasia or infectious organisms is detected on the examined sections.

Blood culture for both aerobic and anaerobic organisms and an antinuclear antibody test performed to differentiate other infectious or immune-mediated conditions yielded negative results. A protein electrophoresis test was also conducted to determine whether the condition was attributable to a congenital or acquired immune response or neoplastic proliferation, and the results showed an elevated alpha-2 globulin fraction, a non-specific result. Cytological evaluation of the synovial fluid revealed a marked increase in hypersegmented neutrophils, with no evidence of pathogens or phagocytosis (Figure 2A). Inflammatory cells, including neutrophils and plasma cells, were identified in the bone tissue obtained from the distal femur (Figure 2B). Histopathological examination showed moderate numbers of histiocytes, clusters of degenerate neutrophils, occasional multinucleated giant cells, and scattered lymphocytes and plasma cells (Figure 3). These cytological and histological findings are suggestive of osteomyelitis. No pathogens were detected in the microbial cultures of the synovial fluid and distal femur bone tissue.

Carprofen (2.2 mg/kg, PO, q12h) and amoxicillin/clavulanate (13.75 mg/kg, PO, q12h) were empirically prescribed beginning on Day 5 of presentation. Antibiotic therapy (amoxicillin/clavulanate) was discontinued on Day 12 following negative culture results. Given the absence of infection or neoplasia in the histological and cytological findings, a diagnosis of CNO was made on Day 17, and the treatment plan was adjusted accordingly. In accordance with human CNO treatment protocols, pamidronate (2 mg/kg, IV, once monthly) and sulfasalazine (15 mg/kg, PO, q8h) were initiated on the day of diagnosis. On the same day (Day 17), carprofen was discontinued, and prednisolone (1 mg/kg, PO, q12h) was introduced three days later (Day 20) following a washout period.

On Day 31, which corresponded to the 2-week follow-up after the diagnosis of CNO, no radiographic improvement was observed. However, partial weight-bearing and marked gait improvement were noted. Consequently, the prednisolone was tapered, with a 25% dose reduction every two weeks.

**Figure 4:**
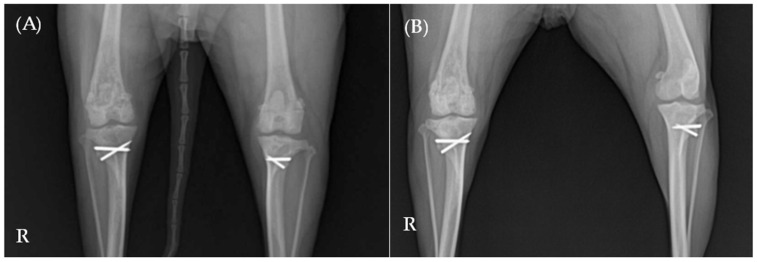
Ventrodorsal radiographic examination of the hindlimb. (**A**) On Day 47, the radiographs showed significant improvement. (**B**) On Day 157, further radiographic improvements were evident, including increased bone opacity and continued reduction in periosteal reaction. The letter ‘R’ indicates the right side of the patient.

On Day 47, at the 1-month follow-up after diagnosis, radiographic evaluations revealed significant improvements (Figure 4A). Muscle thickness in the right femur had increased, bone opacity in the right distal femur and proximal tibia had improved, and the periosteal reaction was notably reduced. At this stage, the patient exhibited a fully normalized gait (Supplementary Video S2), and CRP levels had returned to the reference range.

The patient was subsequently transferred to the local veterinary clinic at the request of the owner, due to geographic distance and convenience of follow-up visits. Although long-term combination therapy with the three prescribed drugs was recommended, pamidronate and sulfasalazine were discontinued at the local clinic on Day 76 (2-month follow-up after diagnosis) due to the owner’s financial constraints in the case of pamidronate and a suspected worsening of keratoconjunctivitis sicca (KCS)—a known adverse effect associated with sulfasalazine.

According to follow-up records from the local veterinary clinic, prednisolone was gradually tapered from 1 mg/kg PO q12h (initiated on Day 20) to 0.25 mg/kg PO q48h by Day 75, following gait improvements. On Day 96, three weeks after the dose reduction, the first relapse occurred, presenting as non-weight-bearing lameness. The prednisolone dose was immediately increased to 0.5 mg/kg PO q12h on Day 96, resulting in clinical improvement within three days.

The prednisolone dose was subsequently tapered again to 0.25 mg/kg PO q24h by Day 118. However, a second relapse occurred on Day 131, two weeks after the dose reduction. On this occasion, increasing the dose to 0.25 mg/kg PO q12h again led to symptomatic improvement within three days.

On Day 157, following further radiographic and clinical improvements—including enhanced bone opacity, reduced periosteal reaction (Figure 4B), and a return to normal gait—the prednisolone dose was tapered to 0.25 mg/kg PO q24h.

At the 6-month follow-up, based on telephone communication with the local veterinary clinic, it was confirmed that the patient was experiencing another relapse at the time of contact, which had occurred approximately three weeks after the most recent dose reduction on Day 157. At that time, the patient was being managed with prednisolone monotherapy, and the dosage was being actively adjusted in response to the recurrence of clinical signs. Unfortunately, no further follow-up could be conducted beyond this point, as the patient did not return to either our hospital or the local veterinary clinic.

## 3. Discussion

Only a few cases of non-infectious osteomyelitis have been reported in veterinary medicine [6,7,8]. This may reflect both the rarity of the disease and the challenges inherent in its diagnostic process. Typically, diagnosis is conducted through exclusion, requiring a comprehensive evaluation that includes clinical signs, laboratory examinations, diagnostic imaging, cytology, and histopathology [2].

CNO presents a range of clinical manifestations, from asymptomatic or mild single-bone lesions to severe local pain in multifocal bone lesions [2]. In the human literature, CNO can occur in various bones, most commonly in the anterior chest wall, clavicle, and epiphyses of long bones [2,9]. In this case, the patient exhibited lesions in the right stifle, leading to non-weight-bearing lameness, warmth, pain, and an aggressive response upon limb examination.

Unremarkable findings in laboratory examinations often characterize CNO; however, elevated inflammatory markers are occasionally observed [10]. Some studies have shown that elevated inflammatory markers are significantly associated with the number of affected sites and the severity of lesions [11,12]. In this case, the CRP level was nine times above the upper limit, indicating severe lesions. The increase in ALP was likely attributed to elevated bone ALP, and no other significant abnormalities were identified.

Radiographs of CNO patients typically show sclerotic, lytic, or mixed lesions, although they are not specific for the diagnosis of CNO [2]. These findings can also be observed in bacterial osteomyelitis, malignant bone tumors, benign bone tumors, and hematological malignancies, making a differential diagnosis essential [3]. Differentiating between inflammatory bone lesions—whether infectious, non-infectious, or neoplastic—on plain radiographs can be challenging, so a definitive diagnosis often requires histopathological analysis and culture.

Advanced imaging modalities such as magnetic resonance imaging (MRI) and computed tomography (CT) play crucial roles in diagnosing CNO in human medicine. MRI can detect early exacerbation of bone edema followed by osteolytic or sclerotic lesions [2]. CT effectively depicts these osteolytic or sclerotic changes, and in later stages, MRI can identify periosteal and soft tissue involvement in human patients [2]. Recent proposals in human medicine also suggest that a bone biopsy may not be necessary if MRI reveals multiple lesions in characteristic locations (such as the clavicle, metaphyses of long bones, and vertebral bodies) in conjunction with normal laboratory results and the absence of systemic symptoms [13]. While similar imaging findings (such as bone marrow edema, osteolysis, and osteosclerosis) may occur in veterinary cases [14,15], CNO incidence in animals is significantly lower than in humans, and documented instances remain limited. Thus, additional case reports and research are necessary to validate the diagnostic value of MRI and CT as reliable diagnostic tools for veterinary CNO.

In our patient, osteolysis and periosteal reaction were observed in the radiograph of the right hindlimb. A bone biopsy was conducted to definitively diagnose the lesion, with cytological and histological findings confirming CNO. Due to the owner’s financial constraints and the uncertain diagnostic value of MRI and CT for CNO in veterinary patients, these imaging modalities were not performed. This approach ensured accurate diagnosis while considering economic factors.

A definitive treatment protocol for CNO has yet to be established in both human and veterinary medicine. However, various therapeutic approaches are currently under investigation in human medicine. NSAIDs (such as naproxen 375–1100 mg/day and celecoxib 200–400 mg/day) administered for 2–4 weeks are commonly used as the first-line treatment in human medicine [5,9]. However, initial NSAID therapy with meloxicam (0.2 mg/kg PO q24h) for 2 weeks, prescribed at the local veterinary clinic, was ineffective. After a 5-day washout period, carprofen (2.2 mg/kg PO q12h) was initiated on Day 5 of presentation to our hospital and administered for one week. Despite these treatments, the patient showed no clinical improvement, necessitating the use of second-line medications typically employed in human medicine. Human consensus guidelines for spinal CNO recommend adding bisphosphonates or DMARDs if no improvement is observed after a minimum of NSAID therapy [5], and the treatment of our patient was guided by this recommendation.

In the human literature, bisphosphonates such as pamidronate specifically target bone osteoclastic activities, making them preferable over other analgesics in bone-related diseases [2,3,16]. Although the exact mechanism of action remains unclear, bisphosphonates can reduce the development of osteoclast precursor cells and promote the apoptosis of mature osteoclasts [2,3,16]. In particular, pamidronate has demonstrated significant efficacy in pediatric patients with CNO refractory to NSAIDs, with multiple studies reporting pain relief, improved quality of life, and resolution of bone lesions without serious adverse effects [2,5]. Bisphosphonates in veterinary medicine are similarly utilized for managing refractory hypercalcemia, providing palliative adjunct analgesia for osteosarcoma-related bone pain, and treating osteoarthritis in horses [17,18,19]. Reported adverse effects of bisphosphonates in humans most commonly include flu-like symptoms; however, atypical subtrochanteric femur fractures and osteonecrosis of the jaw have also been documented in rare instances [2,20]. Oral mucosal ulceration has been reported in dogs, highlighting the need for close monitoring during bisphosphonate therapy [20]. In the present case study, despite the absence of hypercalcemia, pamidronate was administered to mitigate CNO-related pain and osteoclastic activity. Notably, no adverse effects were observed throughout the course of treatment.

Another alternative when NSAIDs are ineffective is DMARDs, including sulfasalazine and methotrexate [5]. DMARDs are a group of medications commonly used in autoimmune diseases such as rheumatoid arthritis, suppressing overactive systemic immune and/or inflammatory responses [5]. In veterinary medicine, sulfasalazine is primarily employed as an adjunctive treatment for autoimmune diseases such as inflammatory significant bowel disease [21]. This case represents the first report of using sulfasalazine as a DMARD for treating CNO, demonstrating favorable therapeutic outcomes. Therefore, it is a potential treatment option for CNO cases and could be extended to other autoimmune diseases.

Lastly, glucocorticoids can be combined with bisphosphonates and DMARDs to treat CNO at a short-term dosage of 2 mg/kg, q24h [3,5]. Low-dose prednisolone (0.1–0.2 mg/kg, PO, q24h) is sometimes utilized as bridging therapy to manage symptoms temporarily until the concurrently administered DMARDs reach their full therapeutic effect [3]. Glucocorticoids regulate bone inflammation by inhibiting phospholipase A1, thereby reducing prostaglandin production and suppressing the expression of pro-inflammatory cytokines such as IL-1, IL-6, and TNF-α through NFκB transcription factor modulation [3]. In veterinary medicine, glucocorticoids are first-line drugs for most immune-mediated diseases [22]. In this case, prednisolone (1 mg/kg, PO, q12h) was similarly administered as a glucocorticoid to mitigate bone inflammation and serve as bridging therapy until the DMARDs achieved full therapeutic efficacy with no adverse effects observed.

Following diagnosis, long-term combination therapy with gradual tapering—comprising pamidronate, sulfasalazine, and prednisolone—was recommended. However, after transfer, the owner elected to discontinue pamidronate due to financial constraints, as the clinical signs had markedly improved. Sulfasalazine was also discontinued due to a suspected exacerbation of KCS, a known adverse effect associated with this drug.

As a result, treatment beyond Day 76 was limited to prednisolone monotherapy. Although the patient demonstrated clinical improvement following dose escalation during relapse episodes—suggesting that prednisolone alone may provide short-term symptomatic relief—its ability to achieve sustained disease control remains uncertain. At the 6-month follow-up, telephone communication with the local veterinary clinic revealed a waxing and waning clinical course despite continued prednisolone administration, indicating that monotherapy may not have been sufficient to consistently suppress disease activity. Furthermore, given the multifactorial pathophysiology of CNO (including immune dysregulation, osteoclastic activation, and cytokine-mediated bone inflammation), it is plausible that targeting a single pathway with glucocorticoids alone may be inadequate in some cases.

In human medicine, both bisphosphonates and DMARDs are typically administered and monitored for a duration of 6 to 12 months to achieve durable disease control in CNO [5]. Given that each of the three agents exerts therapeutic effects via distinct mechanisms, combination therapy may provide synergistic benefits. Although the physiological basis and clinical relevance of such synergistic effects have not yet been elucidated in dogs, it is possible that sustained combination therapy could have resulted in more favorable outcomes with respect to relapse prevention in this case. Therefore, further studies are warranted to investigate the individual and combined efficacy of these agents in the management of canine CNO.

Similar relapse patterns have been frequently reported in human CNO, with relapse rates reaching up to 16% within the first year of diagnosis [3]. Such a recurring activity is a hallmark of the condition, and intervals between recurrences extending up to 6 years have been reported [2]. Given the rarity of CNO, accurately predicting relapse trends is challenging, highlighting the importance of extended clinical monitoring.

A key limitation of this case is the lack of long-term follow-up data beyond six months. The patient did not return to either our hospital or the local veterinary clinic after the 6-month follow-up, thereby precluding further clinical monitoring and assessment of sustained treatment response.

## 4. Conclusions

To our knowledge, this is the first documented case of the medical management of CNO in a dog. Following the diagnosis of CNO, a human treatment protocol targeting its immune-mediated nature, including bisphosphonate, DMARDs, and glucocorticoids, was successfully implemented, yielding favorable clinical outcomes in combination therapy. Our case highlights that adapting human treatment strategies may be beneficial, and further research is needed to clarify the diagnostic criteria and pathophysiology of CNO in veterinary medicine.

## Data Availability

The original contributions presented in the study are included in this article; further inquiries can be directed to the corresponding author.

## Supplementary Materials

The following supporting information can be downloaded at: https://www.mdpi.com/article/10.3390/ani15111593/s1, Video S1: Gait on the Day of Presentation; Video S2: Gait on Day 47 of presentation.

## Abbreviations

The following abbreviations are used in this manuscript:

CNO	Chronic nonbacterial osteomyelitis
CRMO	Chronic recurrent multifocal osteomyelitis
CT	Computed tomography
IV	Intravenous
MRI	Magnetic resonance imaging
NSAIDS	Nonsteroidal anti-inflammatory drugs
PO	Oral administration
